# Exploration of 2D and 3D-QSAR analysis and docking studies for novel dihydropteridone derivatives as promising therapeutic agents targeting glioblastoma

**DOI:** 10.3389/fphar.2023.1249041

**Published:** 2023-08-31

**Authors:** Meichen Pan, Lingxue Cheng, Yiguo Wang, Chunyi Lyu, Chao Hou, Qiming Zhang

**Affiliations:** ^1^ First Clinical Medical College, Shandong University of Traditional Chinese Medicine, Jinan, China; ^2^ Department of Gastroenterology, 960th Hospital of the Chinese People’s Liberation Army, Jinan, China; ^3^ Medical Laboratory Center, Chinese Academy of Traditional Chinese Medicine, Beijing, China

**Keywords:** glioblastoma, drug design, dihydropteridone derivatives, QSAR, molecule docking

## Abstract

**Background:** Dihydropteridone derivatives represent a novel class of PLK1 inhibitors, exhibiting promising anticancer activity and potential as chemotherapeutic drugs for glioblastoma.

**Objective:** The aim of this study is to develop 2D and 3D-QSAR models to validate the anticancer activity of dihydropteridone derivatives and identify optimal structural characteristics for the design of new therapeutic agents.

**Methods:** The Heuristic method (HM) was employed to construct a 2D-linear QSAR model, while the gene expression programming (GEP) algorithm was utilized to develop a 2D-nonlinear QSAR model. Additionally, the CoMSIA approach was introduced to investigate the impact of drug structure on activity. A total of 200 novel anti-glioma dihydropteridone compounds were designed, and their activity levels were predicted using chemical descriptors and molecular field maps. The compounds with the highest activity were subjected to molecular docking to confirm their binding affinity.

**Results:** Within the analytical purview, the coefficient of determination (R^2^) for the HM linear model is elucidated at 0.6682, accompanied by an R^2^_cv_ of 0.5669 and a residual sum of squares (S^2^) of 0.0199. The GEP nonlinear model delineates coefficients of determination for the training and validation sets at 0.79 and 0.76, respectively. Empirical modeling outcomes underscore the preeminence of the 3D-QSAR model, succeeded by the GEP nonlinear model, whilst the HM linear model manifested suboptimal efficacy. The 3D paradigm evinced an exemplary fit, characterized by formidable Q^2^ (0.628) and R^2^ (0.928) values, complemented by an impressive F-value (12.194) and a minimized standard error of estimate (SEE) at 0.160. The most significant molecular descriptor in the 2D model, which included six descriptors, was identified as “Min exchange energy for a C-N bond” (MECN). By combining the MECN descriptor with the hydrophobic field, suggestions for the creation of novel medications were generated. This led to the identification of compound 21E.153, a novel dihydropteridone derivative, which exhibited outstanding antitumor properties and docking capabilities.

**Conclusion:** The development of 2D and 3D-QSAR models, along with the innovative integration of contour maps and molecular descriptors, offer novel concepts and techniques for the design of glioblastoma chemotherapeutic agents.

## 1 Introduction

Glioblastoma (GBM), as a highly malignant tumor, originates from genetic alterations in neural glial stem cells or progenitor cells, rendering it highly invasive and lethal (Wang and Jiang, 2013; Schiff et al., 2019). Clinical manifestations of GBM primarily include increased intracranial pressure, seizures, headaches, and neurological deficits (Márquez et al., 2017; Doan et al., 2020). The current standard treatment for GBM is surgical resection. However, due to the highly infiltrative nature of GBM, the active region often overlaps extensively with vital brain areas involved in motor function and language, making complete eradication through surgery challenging and leading to disease progression and recurrence (Davis, 2016). Approximately 70% of GBM patients experience disease progression within a year after diagnosis, with less than 5% surviving beyond 5 years post-diagnosis (Zhu et al., 2019). Consequently, multimodal therapies such as radiation therapy and immunotherapy are often concurrently employed alongside surgical intervention (Tan et al., 2020).

Currently, several chemotherapy drugs are available for GBM treatment, including temozolomide, bevacizumab, carboplatin, etoposide, and irinotecan (Zhao et al., 2020). However, these drugs are associated with adverse effects such as bleeding, perforation, and hepatorenal dysfunction. Prolonged use of a single type of chemotherapy drug also leads to the development of drug resistance, further compromising patient prognosis (Wu et al., 2021). Nonetheless, the molecular mechanisms underlying GBM recurrence, metastasis, drug resistance, and toxicity remain incompletely elucidated. Limited progress has been made in chemotherapy drug research since 2005 (Jackson, Choi, and Lim, 2019). Consequently, there is a pressing need to develop GBM chemotherapy drugs with reduced toxicity and improved efficacy to enhance treatment outcomes.

The principal focal point of action for dihydropteridone derivatives lies in Polo-like kinase 1 (PLK1). PLK1 assumes a pivotal role in numerous functions, encompassing DNA checkpoint regulation, cellular division, microtubule dynamics, and DNA replication/repair (Kim et al., 2017). Being a proto-oncogene, PLK1 expression levels are significantly elevated in various malignancies, including glioblastoma, making it a potential therapeutic target for glioblastoma treatment (Bhola et al., 2015). Dihydropteridone derivatives exert their anticancer effects primarily by interfering with folate metabolism and inhibiting the dihydropteridone reductase pathway (Martin et al., 2020). By impeding the synthesis of nucleotides, the building blocks of DNA and RNA, these drugs disrupt fundamental processes involved in tumor cell development and proliferation (Hofheinz et al., 2010). Additionally, they can induce DNA damage and promote apoptosis in tumor cells (Kim et al., 2017). Moreover, the synthesis process of dihydropteridone-class compounds is relatively straightforward, and their production costs are not exorbitant, providing a foundation for their future prospects in anticancer treatment. Recently, a novel dihydropteridone derivative has garnered attention (Li et al., 2023), as it possesses the aforementioned advantages and also incorporates an oxadiazole moiety, significantly ameliorating the inherent metabolic vulnerability of amides to hydrolysis by esterases and hepatic amidases (Fukami and Yokoi, 2012; Robins, Fogle, and Marlier, 2015). This enhancement in metabolic stability contributes to its improved anticancer activity and opens new avenues for designing chemotherapy drugs targeting glioblastoma.

To facilitate the efficient design and evaluation of novel drugs, we introduce computer-aided drug design, with quantitative structure-activity relationship (QSAR) being the most exceptional experimental approach (Janicka and Śliwińska, 2022). This mathematical framework establishes a correlation between the scrutiny of structural attributes and the corresponding pharmacological efficacy. In previous studies on QSAR modeling, the 2D model primarily focuses on elucidating the impact of the molecular descriptors’ quantity and class on drug activity. Conversely, the 3D model places emphasis on exploring the correlation between the spatial configuration of the molecule and its activity. In the present study, we aim to leverage the strengths of both approaches—molecular descriptors and molecular force fields—to develop a predictive model for the activity of dihydropteridone derivatives against GBM. By employing this mathematical model, our objective is to facilitate the design of more efficacious chemotherapeutic drugs targeting GBM.

## 2 Materials and methods

### 2.1 Data set acquisition

All the dihydropteridone derivatives with an oxadiazole moiety used in this experiment were obtained from the research conducted by Zhiwei Li et al. (Li et al., 2023). The structures and corresponding activity values of the 34 compounds are presented in Table 1.

**TABLE 1:** Structure and IC_50_ values of 47 compounds.

Structure	Substituent	IC50 (µM)	No
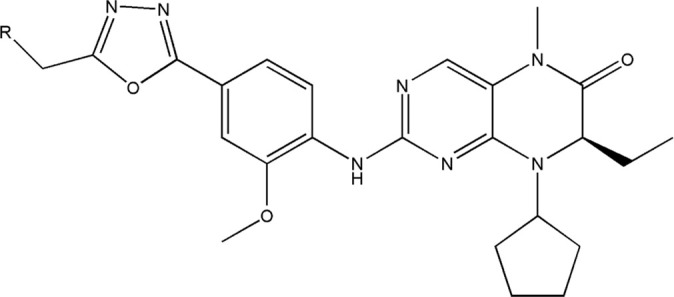	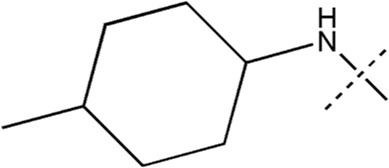	0.3	13a^a^
	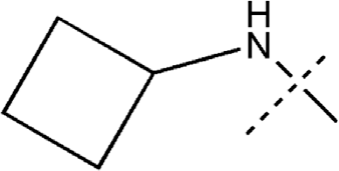	0.53	13b
	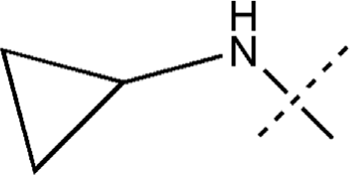	0.19	13c
	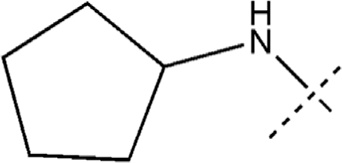	0.4	13d^ays^ ^a^
	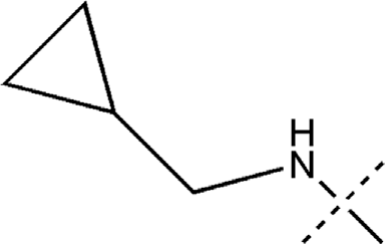	0.39	13e
	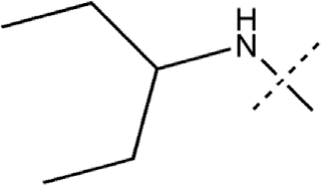	0.69	13f
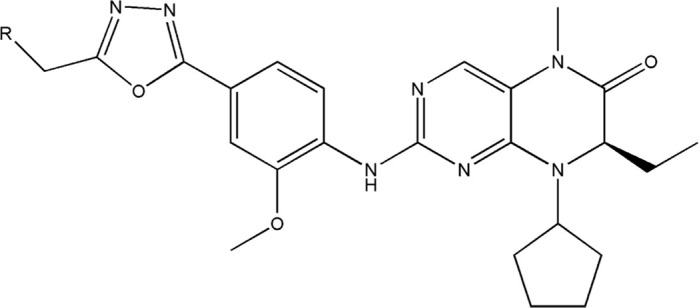	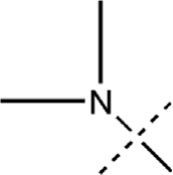	0.23	13g
	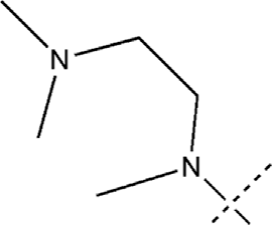	0.64	13h^a^
	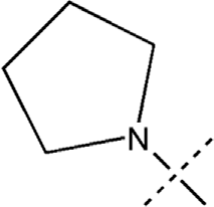	0.6	13i
	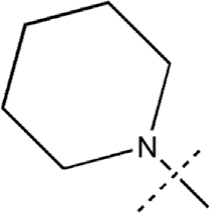	0.62	13j
	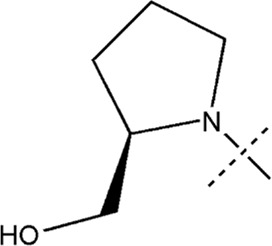	0.23	13k
	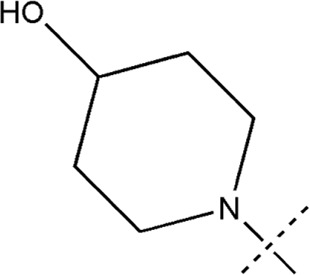	0.55	13l
	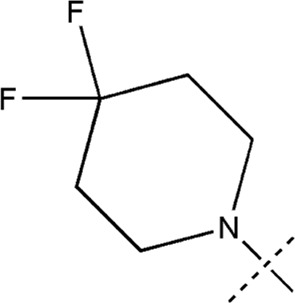	1.07	13m^a^
	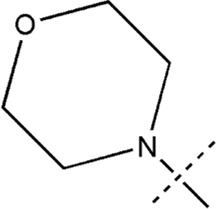	0.42	13n
	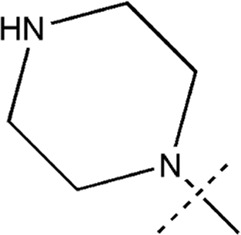	0.33	13o
	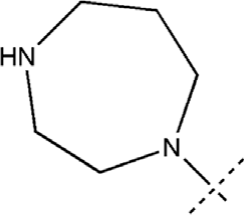	0.42	13p
	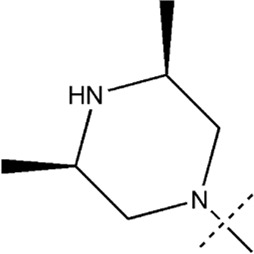	0.5	13q
	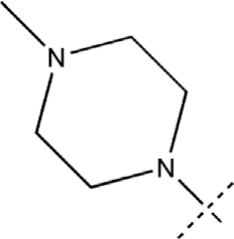	0.79	13r
	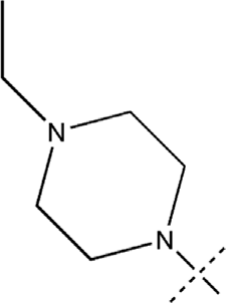	0.63	13s^a^
	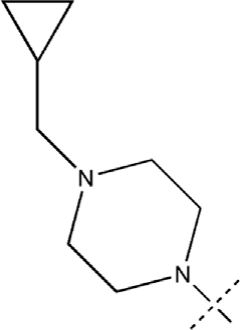	0.53	13t
	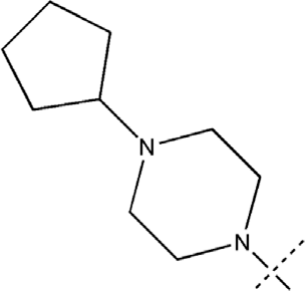	0.34	13u
	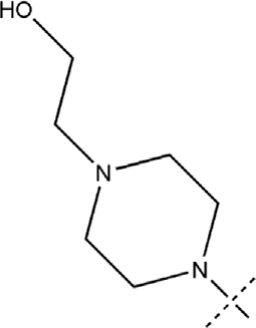	0.44	13v
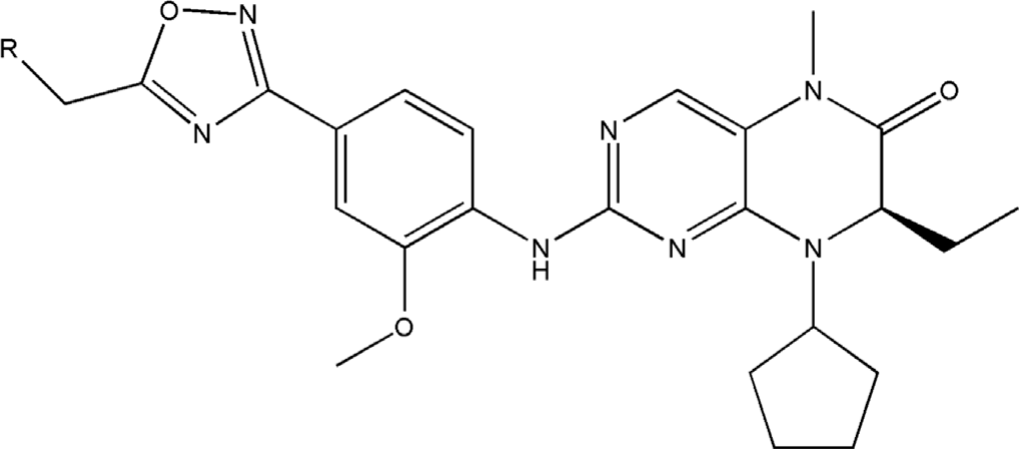	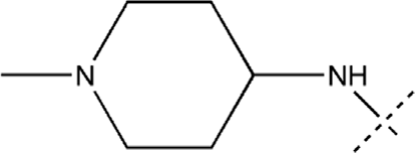	0.62	21a
	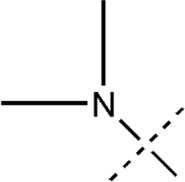	1.02	21b
	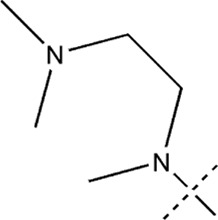	0.53	21c
	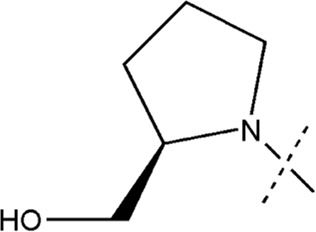	0.5	21d ^a^
	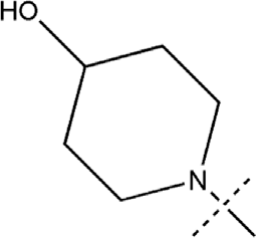	0.18	21e
	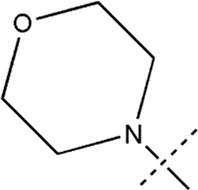	0.76	21f
	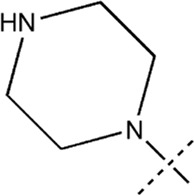	0.45	21g
	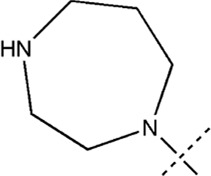	0.32	21h
	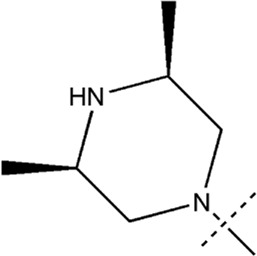	0.37	21i^a^
	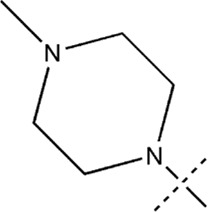	0.91	21j
	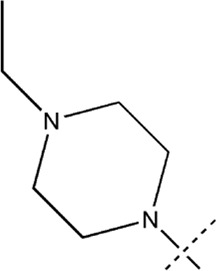	0.55	21k
	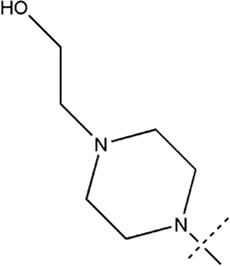	0.26	21l^a^

Note:In the 2D-QSAR, experiment, the test set is denoted by.

^a^ , while in the 3D-QSAR, experiment, it is indicated by underlining.

### 2.2 Exploring 2D-QSAR analysis

#### 2.2.1 Handling of 2D-QSAR dataset

To mitigate the risk of overfitting, a random partitioning was applied to the set of 34 compounds at a ratio of 1:3, resulting in 8 compounds assigned to the test set and 26 compounds allocated to the training set. The primary objective of the training set is to establish and refine the model, encompassing the construction, calibration, as well as the identification of key variables and algorithms. Meanwhile, the test set serves primarily for model calibration purposes, ensuring the assessment remains unbiased and does not involve parameter modification. Ultimately, decisions regarding algorithm adjustments or model retraining are contingent upon evaluating the overall fit of the model.

#### 2.2.2 Selection of molecular descriptors and refinement of compounds

The performance of the QSAR model relies heavily on the appropriate selection of molecular descriptors, necessitating the structural optimization of the compound under investigation. In this study, the chemical structure was initially sketched using ChemDraw (Evans, 2014) and subsequently subjected to optimization using HyperChem (Froimowitz, 1993). The optimization process involved employing the molecular mechanics field (MM+) for the initial optimization, followed by the selection of the AM1 or PM3 model based on the presence or absence of S and P atoms. Furthermore, the structure was cyclically optimized using the Polak-Ribiere method until the root mean square gradient reached a threshold of 0.01. Finally, the CODESSA program (Katritzky et al., 2001) was utilized to compute molecular descriptors encompassing quantum chemistry, structure, topology, geometry, and electrostatic properties.

#### 2.2.3 Heuristic construction of linear models

In the process of constructing linear models, the Heuristic Method (HM) was employed to extract all molecular descriptors. Subsequently, feature selection was conducted to determine the optimal number of descriptors that effectively represent the chemical structure while excluding descriptors with minimal impact. Objective measures, such as the F-test, R^2^, R^2^_CV_, and t-test, were used to evaluate the correlation coefficients between two parameters. Additional descriptors were iteratively added until further inclusion of descriptors had little influence on the results. The linear model obtained through this procedure consisted of six descriptors.

#### 2.2.4 Development of nonlinear models utilizing GEP

Gene Expression Programming (GEP) is a powerful technique rooted in programming and algorithms (Allen, 1992), surpassing the capabilities of both. Unlike coding numbers or analyzing trees, GEP utilizes linear chromosomes as candidates (Kaydani, Mohebbi, and Eftekhari, 2014). The coding of constant-length linear symbols and the derivation of individual phenotypes, similar to coding codes and expression trees, respectively, are employed (Teodorescu and Sherwood, 2008). The candidate chromosomes are generated from the feature set and the end set, and then encoded into an expression tree (ET) format to calculate the equation (Gharagheizi et al., 2012).

**FIGURE 1:**
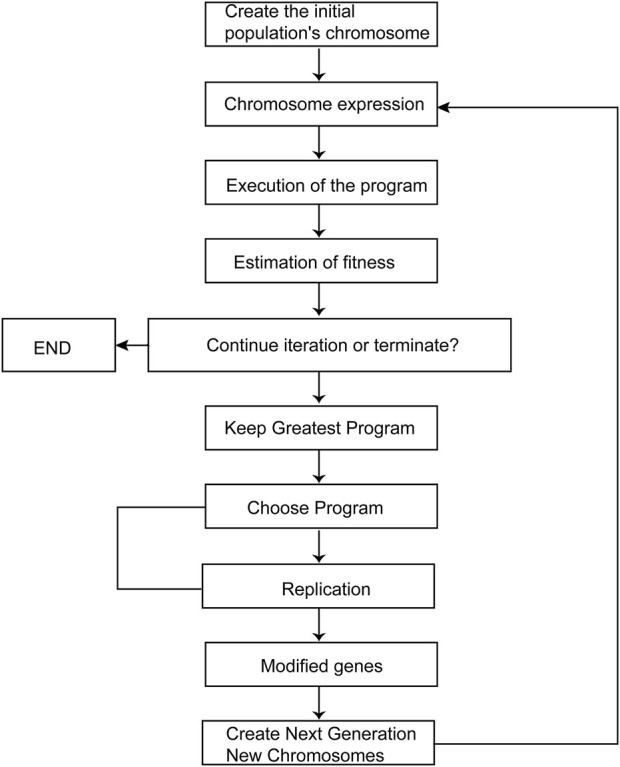
GEP diagram.

Figure 1 illustrates the overall process of GEP. Fitness functions are applied to a random number of chromosomes, with termination conditions being either the achievement of the predicted value or reaching the maximum number of iterations. When the termination requirement is not met, individuals are selected using the elite roulette approach. Genetic operations such as mutation, transposition, and recombination are applied to the selected individuals to form a new generation. This process is repeated iteratively to obtain improved results.

At the operational level, the molecular descriptor values are inputted using automated problem software (APS), and the GEP technique is employed to derive the nonlinear model. The quality of the model is evaluated using R^2^ as an objective assessment indicator, and appropriate parameter selection is critical to achieve this objective.

Thus, two models were designed: one linear and the other nonlinear. Clearly, the nonlinear model generated by the GEP method outperforms the linear model in terms of both predictiveness and stability. However, it should be noted that 2D-QSAR alone fails to fully capture the three-dimensional relationship between molecular structure and activity, highlighting the need for further exploration through 3D-QSAR studies.

### 2.3 The exploration of 3D-QSAR

#### 2.3.1 Data manipulation and structural refinement

The IC_50_ denotes the concentration of a compound necessary to inhibit a biological process by 50% (Sebaugh, 2011), and it frequently covers a wide range of magnitudes. In light of this, we employed the formula log (IC_50_) + 9 to substitute the IC_50_ values for the 34 compounds. This approach was undertaken to facilitate the analysis and processing of the data, ultimately enhancing both accuracy and stability. These modified data were randomly divided into validation and training sets in a 1:4 ratio.

Furthermore, the ChemDraw structures of the compounds were imported into the SYBYL program for additional optimization. Unlike previous optimizations, this step aimed to minimize the energy of the CoMSIA structure as much as possible. To achieve this, the Tripos force field and Powell gradient technique were implemented within the software (Yu et al., 2015).

This optimization process was underpinned by a systematic calibration of salient operational parameters. The Dielectric Function was set to “Distance,” the MB Cut-off was designated at 8, and the Dielectric Constant was adjusted to 1. Further refinement was achieved by determining the Max Displacement at 0.01, establishing the Minimum Energy Change at 0.05, setting the LS Accuracy to 0.001, configuring the RMS Displacement at 0.001, and specifying the Gradient at 0.05. Following this intricate calibration, the software initiated a comprehensive sequence of 1,000 iterations, leading to the generation of an optimal minimal structure. This resultant structure subsequently served as the foundational 3D conformation for ensuing analytical stages.

#### 2.3.2 Conformation comparison and selection

**FIGURE 2:**
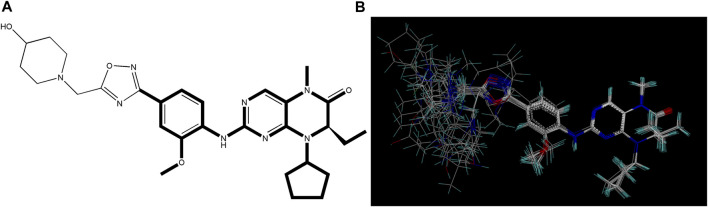
Utilization of compound 21E as a molecular stacking template. **(A)** Bold highlighting indicates the stacking regions shared by all compounds. **(B)** Graph illustrating the subsequent stacking process.

The selection and comparison of conformations are crucial as the compound’s structure plays a vital role in subsequent modeling processes (Patel et al., 2008; Ai et al., 2011). Among the 34 compounds, 21E, which exhibited the highest activity value, was chosen as the reference or stacking template. The bold segment of 21E is exceptionally designated due to its manifestation of the identical structure that compound 21E shares with the other compounds. Based on this reference, the remaining compounds were aligned and arranged accordingly (as shown in Figure 2).

#### 2.3.3 CoMSIA research

The Comparative Molecular Similarity Index Analysis (CoMSIA) is based on the concept that changes in molecular bond affinities are strongly correlated with changes in molecular properties (Yu et al., 2015). This technique involves the calculation of molecular fields as numerous contour plots using a Gaussian function that depends on distance (Li et al., 2012). In contrast to CoMFA, CoMSIA utilizes contour plots to depict the five distinct spatial molecular fields. This approach eliminates the need for arduous procedures, such as aligning the grid with the molecules in the dataset, which is required in CoMFA (Bordas, Komives, and Lopata, 2003). As a result, CoMSIA enhances both clarity and precision in the analysis.

The region for molecule stacking is filled with a cubic grid with a pitch of 2 Å and extending 4 Å in all directions. The default probe generates a multi-molecular field grid, and the affinity is associated with molecular properties using partial least squares (PLS). This leads to the construction of a 3D quantitative conformational model.

The leave-one-out (LOO) cross-validation test is employed to determine statistical significance, providing cross-validated correlation coefficients (Q^2^) and best group scores (ONC) (Hadni and Elhallaoui, 2020). Additionally, the ONC is further analyzed through non-cross-validation to formulate the ultimate regression model. Objective assessment indicators such as the standard error of estimate (SEE), F-value, and non-cross-validation correlation coefficient (R^2^) are used for the evaluation of non-cross-validation modeling (Yan et al., 2020).

#### 2.3.4 Verification of the 3D model through external and internal validation

The method of external validation was employed to forecast the activity of the compound set in the test group (Yan et al., 2020), yielding the correlation coefficient $R_{ext}^{2}$ , which was determined by the following equation:

$$R_{ext}^{2}=1-\frac{\sum_{i=1}^{ntest}{\left(y_{i}-{\tilde{y}}_{i}\right)}^{2}}{\sum_{i=1}^{ntest}{\left(y_{i}-{\bar{y}}_{tr}\right)}^{2}}$$

In the aforementioned equation, ntest represents the total quantity of compounds present within the test set. Symbol $\bar{y}tr$ represents the mean value of the compounds’ activity observed within the training set. Additionally, symbols *yi* and $\tilde{y}i$ represent the experimental and predicted values, respectively, of the compounds’ pharmacological activity within the test set. It is worth noting that when the correlation coefficient R^2^ exceeds 0.5, the model demonstrates robustness and exhibits excellent statistical predictive capability (Yang et al., 2011; Mouchlis et al., 2012).

Moreover, we performed an supplementary validation of the model utilizing $R_{m}^{2}$ to assess its rationality (Roy and Mitra, 2012).

The formula is expressed as follows:

$$R_{m\left(overall\right)}^{2}=R^{2}*\left(1-\sqrt{R^{2}-R_{0}^{2}}\right)$$

Within the formula, $R^{2}$ symbolizes the square of the correlation coefficient between the predicted values and experimental values of all compounds in both the test set and validation set, while $R_{0}^{2}$ denotes the square of the correlation coefficient with a zero intercept. When $R_{m}^{2}$ exceeds 0.5, it serves as an indicator of the model’s substantial stability (Pratim Roy et al., 2009).

Meanwhile, an internal validation, specifically, a 20-times Y-randomization validation, was performed to ascertain the optimal model. We subjected the dependent variables to randomization and generated new QSAR models. On each occasion, these newly created models exhibited lower q^2^ and R^2^ values than the original model. Subsequently, the parameter $R_{p}^{2}$ was introduced to assess the disparity between the randomized and original model R^2^. The formula utilized is as follows:

$$R_{p}^{2}=R^{2}*\sqrt{R^{2}-R_{r}^{2}}$$

Where $R_{r}^{2}$ represents the average R^2^ value over the 20 iterations of the stochastic model, and a favorable outcome for the model $R_{p}^{2}$ should yield a value greater than 0.5 (Rücker, Rücker, and Meringer, 2007).

#### 2.3.5 Molecular docking based on the SYBYL software platform

The compounds underwent initial optimization using the SYBYL software, employing the Tripos force field. Following that, the conformation displaying the minimum energy value was chosen for the molecular docking procedure. The PLK1 receptor (ID: 3db6) was obtained from the RCSB Protein Data Bank (PDB) during the second stage. After removing water molecules and hydrogenated atoms, the protein ligand was extracted and discarded, while retaining the binding site for further analysis.

Following this, a flexible docking approach was employed to facilitate the interaction between the ligand and receptor, allowing for the exposure of the active pocket at the binding site. Docking was performed utilizing Sybyl-Dock as the benchmark, employing a docking threshold with a value of 0.5, an expansion factor of 1, and a retention of 20 conformational alterations. The evaluation of ligand-receptor interactions was objectively conducted using a comprehensive scoring function, with higher values indicating a stronger binding impact of the drug. Undoubtedly, the considerations of hydrophobicity, enthalpy, and polarity are crucial in this context.

## 3 Results and discussion

### 3.1 HM-based linear model

**FIGURE 3:**
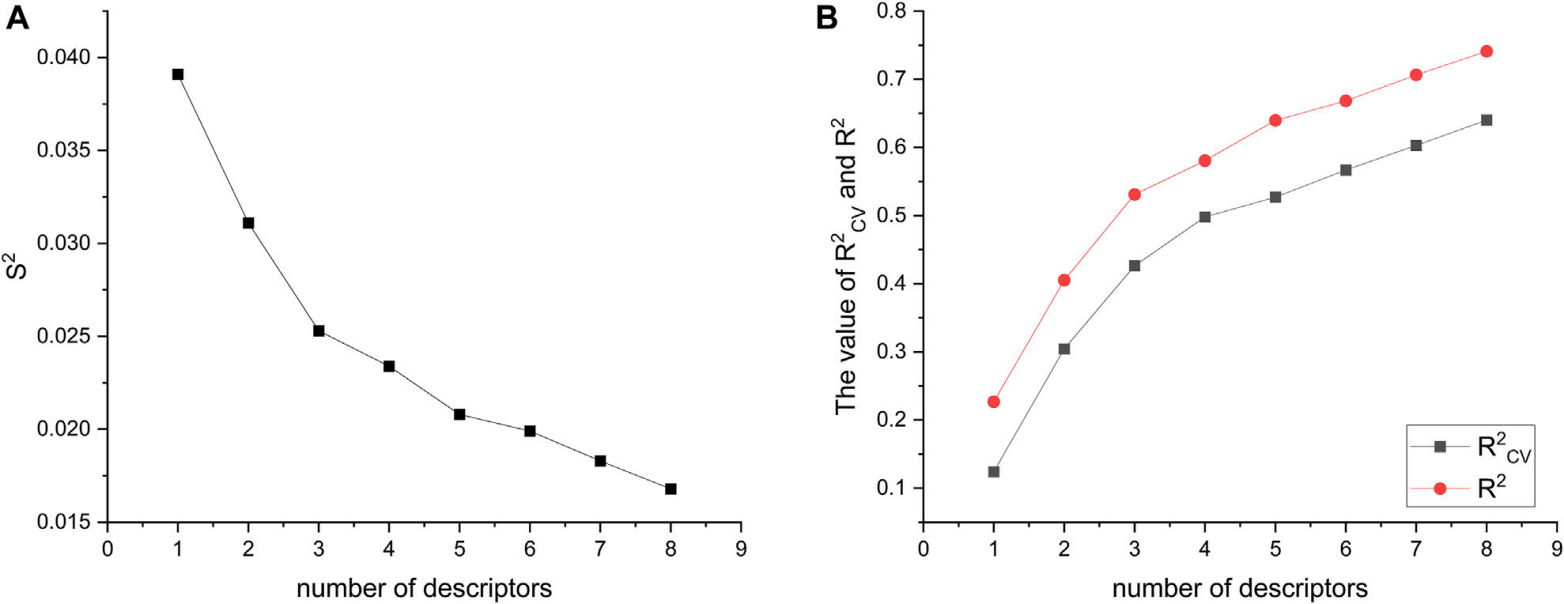
Visualization of descriptor number changes in relation to S^2^ **(A)**, R^2^, and R^2^_cv_ **(B)**.

A total of 501 molecular descriptors were calculated for the 34 compounds using CODESSA. Based on the hierarchical modeling (HM), a linear model consisting of eight distinct descriptor numbers (1–8) was constructed. The relationship between these descriptor numbers and the model’s evaluation indexes, namely R^2^, R^2^_cv_, and S^2^, was examined (refer to Figure 3). It was observed that both R^2^ and R^2^_cv_ showed positive correlations with the increase in descriptor numbers, while S^2^ exhibited an inverse relationship. Notably, the addition of the 7th descriptor did not result in a significant improvement in R^2^. The six-parameter HM model, with an R^2^ value of 0.6682, an R^2^_cv_ of 0.5669, and an S^2^ measuring at 0.0199, is deemed the optimal linear model for evaluating the efficacy of glioma inhibitors.

Specific information about the six modeled molecular descriptors is provided in Table 2. To ensure the absence of multicollinearity, Table 3 presents the correlation between these descriptors. The correlation coefficient between any two descriptors was found to be less than 0.80, indicating their independence and lack of mutual influence. This validates the integrity of the linear model.

**TABLE 2:** Correlation information for the six molecular descriptors.

Symbol	Physical-chemical meaning	Coefficient	*t*-test
NFA	Number of F atoms	2.9798e − 01	4.0171
MRCH	Max e-e repulsion for a C-H bond	4.4403e − 01	2.5384
MECN	Min exchange energy for a C-N bond	5.6298e + 00	3.3342
TEIZP	Topographic electronic index (all bonds) [Zefirov’s PC]	−5.7965e − 01	−3.1843
ZXS	ZX Shadow/ZX Rectangle	3.6953e + 00	2.7395
MCIHN	Min coulombic interaction for a H-N bond	−3.8370e + 00	−1.5254

**TABLE 3:** Correlation table displaying the relationships among the selected six descriptors.

Name	NFA	MRCH	MECN	TEIZP	ZXS	MCIHN
NFA	1	−0.13064	−0.08954	0.00487	0.20426	0.15741
MRCH	−0.13064	1	0.24872	−0.13865	−0.06382	−0.07646
MECN	−0.08954	0.24872	1	−0.04309	−0.02138	0.2345
TEIZP	0.00487	−0.13865	−0.04309	1	0.40439	0.07533
ZXS	0.20426	−0.06382	−0.02138	0.40439	1	0.71179
MCIHN	0.15741	−0.07646	0.2345	0.07533	0.71179	1

**FIGURE 4:**
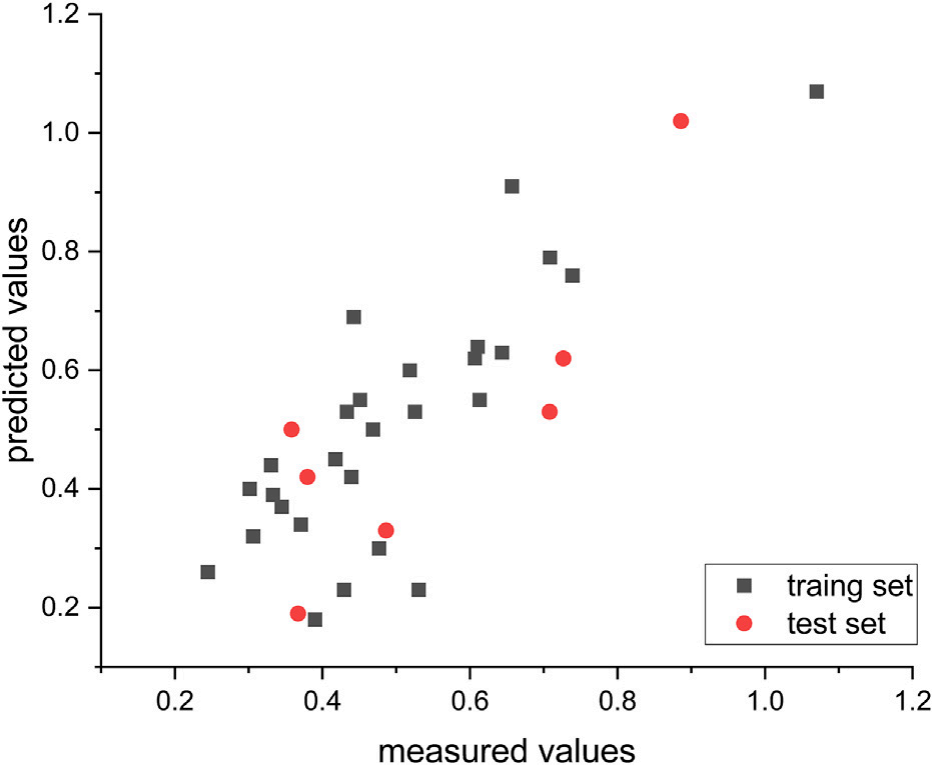
Empirical vs. computed log (IC50) values using the HM methodology.

Figure 4 illustrates the predicted and measured values of the hierarchical modeling (HM) linear model for the compounds. The equation representing this model is as follows: Log (IC_50_) = −2.4280e + 01 + NFA*2.9798e − 01 + MRCH*4.4403e − 01 + MECN*5.6298e + 00 − TEIZP*5.7965e − 01 + ZXS *3.6953e + 00 − MCIHN*3.8370e + 00.

The absolute magnitude of the coefficients was employed to evaluate the impact of the molecular descriptors on the antiglioma activity of dihydropteridone derivatives. It can be observed that the order of influence is as follows: MECN > MCIHN > ZXS > TEIZP > MRCH > NFA.

### 3.2 Developing nonlinear models with GEP

**FIGURE 5:**
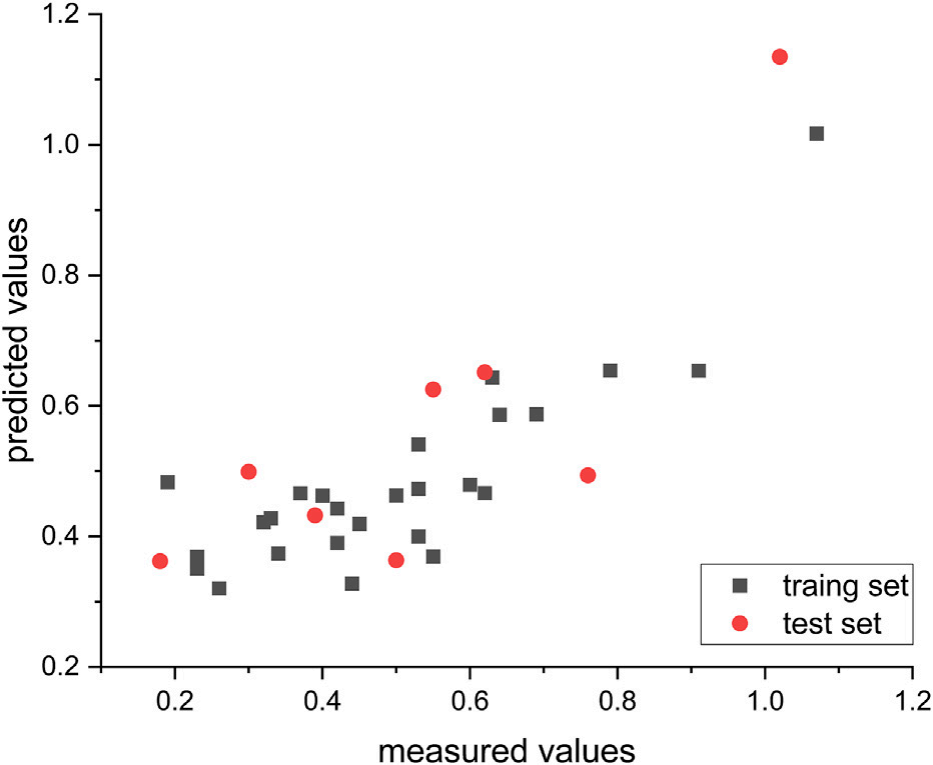
Empirical and predicted values from non-linear algorithms (GEP), with all IC50 measurements converted to their respective log (IC50) notations.

After randomly assigning the 34 compounds, 26 compounds were allocated to the training set, and the remaining 8 compounds were assigned to the test set in proportion. The nonlinear model was constructed using the GEP method implemented in the Automated Problem Solver (APS) program. Table 4 provides the model’s parameters and corresponding symbols. The distribution of predicted and measured values obtained from this model for compound prediction is depicted in Figure 5. The R^2^ values for the training and test sets are 0.79 and 0.76, respectively, indicating a high level of accuracy in the model’s predictions.

**TABLE 4:** Parameters and symbols used in nonlinear equation operations.

Parameter name	Representation	Value
Addition	+	1
Subtraction	−	1
Multiplication	*	1
Division	—	1
Inverse	Inx	1
Sine	Sin	1

The nonlinear model equations were translated into the following representation using the C programming language: Log\[IC_(50) \] = 1\/\(TEIZP − NFA\) + sin\[sin\(TEIZP\) + \(MECN − MECN\)\]\/\(MRCH − MCIHN\) * sin\(MRCH\) + ZXS * 1\/\{MRCH * sin\(ZXS\) \/ZXS + ZXS \/TEIZP + sin\[sin\(sin\(sin\(MRCH\) * sin\(MCIHN\)\)\)\]\}.

### 3.3 The statistical outcome of the CoMSIA study

Table 5 presents the key statistical data for the CoMSIA model with the most optimal performance. The model exhibits high Q^2^ (0.628), R^2^ (0.928), and F-values (12.194), indicating a strong and reliable fit. Additionally, the model demonstrates low SEE values (0.160), further supporting its robustness.

**TABLE 5:** Statistical results of the highly efficient 3D-QSAR model derived from the CoMSIA method.

Model	Q^2^	ONC	R^2^	SEE	F
CoMSIA	0.682	1	0.928	0.160	12.194
Name	S	E	H	D	A
Contribution	0.193	0.194	0.331	0.279	0.218

### 3.4 Results of the external and internal validation

**FIGURE 6:**
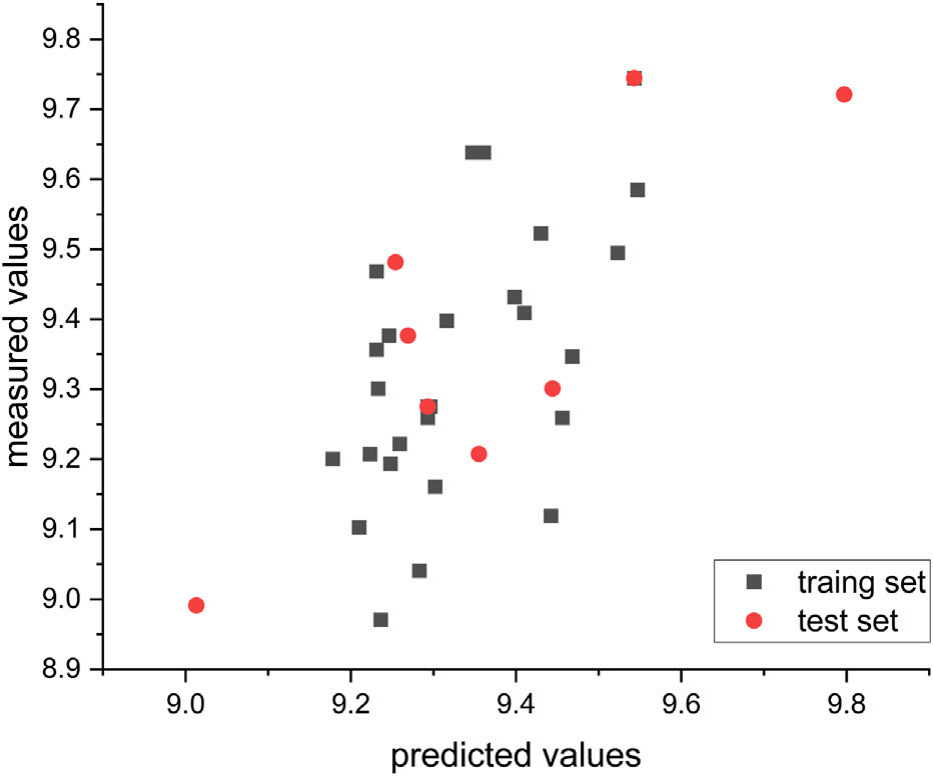
Relationship between measured and predicted values in the CoMSIA model.

When the $R_{ext}^{2}$ , $R_{m}^{2}$ , and $R_{p}^{2}$ values exceed 0.5, the model attains a high level of confidence. Through the analysis utilizing both external and internal validation methods, we acquired an $R_{ext}^{2}$ value of 0.65, an $R_{m}^{2}$ value of 0.64, and an $R_{p}^{2}$ value of 0.61. These findings suggest that the model demonstrates a certain degree of stability and predictability. This finding is supported by Figure 6, which provides visual representation of the model’s predictive capabilities. Presentation of the five molecular fields of CoMSIA.

The utilization of CoMSIA confers a notable advantage wherein it enables the visualization of contour maps pertaining to individual molecular fields (Mao et al., 2012). This feature establishes a fundamental basis for our comprehension of the role of physicochemical structure in activity. Moreover, the identification and annotation of pivotal structures, regions, and active sites have yielded significant advancements in the development of innovative pharmaceuticals.

**FIGURE 7:**
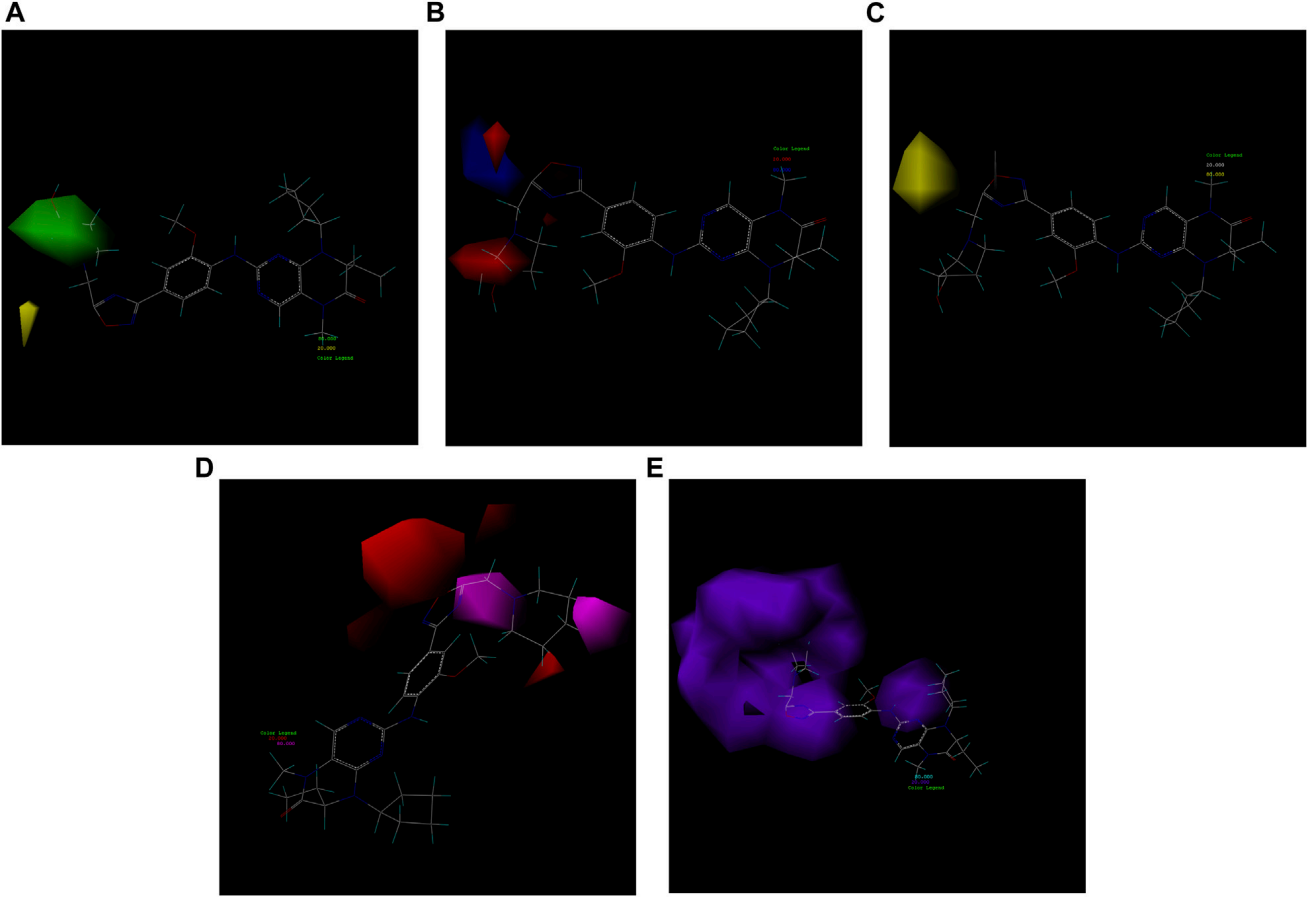
Five molecular field maps of CoMSIA based on the template of compound 21E. **(A)** Steric field, with the increase of the green group positively correlated with activity and the increase of the yellow group negatively correlated. **(B)** Electrostatic field, exhibiting a positive correlation between the increase of the red negative field and blue positive field and the activity. **(C)** Hydrophobic field, where the yellow portion requires strengthening of its hydrophobic structure, while the gray portion necessitates reinforcement of its hydrophilic structure. **(D)** Hydrogen bond acceptor field, indicating the need for an increase in the purple region and a decrease in the red region for hydrogen bond acceptors. **(E)** Hydrogen bond donor field, indicating the need for augmentation in the blue-green region and reduction in the purple region for hydrogen bond donors.

As exemplified in Figure 7, the illustration exemplifies the diverse contributions of the five molecular fields to the most active 21E molecule. Evidently, the hydrophobic field exerts a substantial influence on the compound, thus necessitating a subsequent design orientation in alignment with this aspect.

### 3.5 Designing and predicting the activity of novel compounds

First, we must analyze the regions wherein positive and negative alterations in molecular descriptors lead to augmented or diminished activity, respectively. Subsequently, we shall explore the influence of modifications in the 3D structural attributes within the CoMSIA contour map on the biological activity in 3D space. It is imperative to identify five molecular fields, namely spatial (S), hydrogen bond donor (D), electrostatic (E), hydrogen bond acceptor (A), and hydrophobic (H), where advantageous changes correspond to heightened activity in specific regions. By manipulating the distinct 3D geometries and compositions of the compounds, we can ascertain their association with the favorable regions on the 3D CoMSIA contour map.

Upon evaluating the outcomes of 2D-QSAR analysis on dihydropteridone derivatives, it was determined that the molecular descriptor known as “Minimum exchange energy for a C-N bond” (MECN) exerts the most substantial influence on the compound’s activity. MECN represents the underlying energetics of the C-N bond transformation (Gilli et al., 2002). This energy metric holds paramount importance in the sophisticated realms of molecular interactions and bonding dynamics. It significantly influences the bioactivity of molecular entities by determining their inherent stability, reactivity, and binding propensity (Bariwal and Van der Eycken, 2013). The intrinsic affinity between a molecule and its designated target is central to assessing its therapeutic efficacy. A reduced MECN suggests enhanced stability in molecular engagements with biological entities, implying superior inhibitory responses against pathogenic enzymes or proteins. Conversely, an elevated MECN could compromise these interactions, thereby diminishing a compound’s therapeutic effectiveness. Therefore, understanding and optimizing the MECN is pivotal for amplifying its bioactive potential.

Beyond the MECN, five paramount molecular descriptors are delineated: the “Number of fluorine atoms,” which modulates solubility and metabolic tenacity (Shah and Westwell, 2007); the “Max e-e repulsion for a C-H bond,” articulating molecular conformation and kinetic propensities (Brandes and Ellman, 2022); the “Topographic electronic index (all bonds) [Zefirov’s PC],” epitomizing molecular discernment via electronic topography (Hamlin, Swart, and Bickelhaupt, 2018); the “Min coulombic interaction for a H-N bond,” signifying electrostatic congruencies (Tang et al., 2020); and the “ZX Shadow/ZX Rectangle,” encapsulating the compound’s spatial and electronic disposition (Silakari et al., 2008).

Leveraging this observation, along with the notable importance of the hydrophobic field in CoMSIA, the MECN descriptor was integrated into the design process for novel medications, with a particular emphasis on augmenting the drug’s efficacy through the hydrophobic field.

A total of 200 new dihydropteridone derivatives were synthesized and subsequently assessed using CoMSIA software, utilizing 21E as a template. Due to spatial limitations, only the top eight innovative compounds and their projected activity levels are presented in Table 6. Notably, 21E.153, exhibiting the highest activity value, exhibits promising potential as an anti-glioma medication. Molecular docking experiments were subsequently conducted to further validate our hypothesis.

**TABLE 6:** A Presentation of 200 novel compounds along with their predicted values (shown here are the top eight only).

Name	Predictive value
21E	9.549
21E.33	9.556
21E.4	9.569
21E.38	9.569
21E.13	9.57
21E.37	9.571
21E.2	9.574
21E.40	9.583
21E.153	9.632

### 3.6 Experiments using molecular docking for the most active chemicals

**FIGURE 8:**
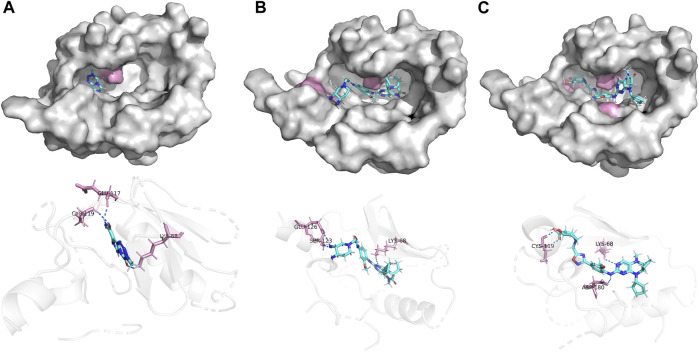
Docking of compounds 21E **(A)**, 21E.153 **(B)**, Temozolomide **(C)** and to a glioblastoma-associated target (PLK1, PDB ID: 3db6).

In this investigation, we employed ligands 21E, 21E.153, and Temozolomide for conducting molecular docking experiments with the glioma-associated target PKL1, respectively. Temozolomide, a commonly utilized chemotherapeutic agent for glioma, was chosen as the docking ligand. Figure 8 illustrates the docking results of these three compounds, wherein the yellow segment denotes hydrogen bonding interactions. It is worth noting that compound 21E.153 exhibits a remarkable presence of three hydrogen bonds, surpassing the quantities observed in compounds 21E and temozolomide. Furthermore, their respective docking scores of 8.7688, 8.7067, and 3.4497 provide additional support for this interpretation. Collectively, the aforementioned observations substantiate the superior docking efficacy of compound 21E.153.

## 4 Conclusion

In this study, our initial approach involved the exploration of both linear and nonlinear 2D-QSAR models. However, it became evident that the nonlinear models outperformed the linear models in terms of stability and prediction capabilities. Nonetheless, both linear and nonlinear models overlook the influence of spatial structure on activity. Hence, we proceeded to develop a 3D-QSAR model utilizing the CoMSIA technique. The robustness of the 3D-QSAR model is demonstrated by its high Q^2^ (0.628), R^2^ (0.928), and F-values (12.194), along with low SEE values (0.160).

Employing the CoMSIA approach not only enables us to uncover the three-dimensional structural variations of the model but also provides us with five molecular field contour maps, which prove to be unexpectedly valuable for the generation of new molecules. By combining the significant molecular descriptor “MECN” with the significant molecular field “hydrophobic field”, we successfully generated and postulated 200 novel compounds. Among these, compound 21E.153 emerged as the most potent.

To validate the affinity of these compounds for glioma-related receptor targets, we conducted molecular docking experiments. Encouragingly, 21E.153 exhibited the most favorable docking interaction, introducing a novel concept and strategy for the future development of glioma medications.

## Data Availability

The datasets presented in this study can be found in online repositories. The names of the repository/repositories and accession number(s) can be found in the article/Supplementary material.
